# The value of multi-phase CT based intratumor and peritumoral radiomics models for evaluating capsular characteristics of parotid pleomorphic adenoma

**DOI:** 10.3389/fmed.2025.1566555

**Published:** 2025-04-22

**Authors:** Qian Shen, Cong Xiang, Yongliang Han, Yongmei Li, Kui Huang

**Affiliations:** ^1^Department of Radiology, The Affiliated Stomatology Hospital of Southwest Medical University, Luzhou, China; ^2^School of Artificial Intelligence, Chongqing University of Technology, Chongqing, China; ^3^Department of Radiology, The First Affiliated Hospital, Chongqing Medical University, Chongqing, China; ^4^Department of Oral and Maxillofacial Surgery, The Affiliated Stomatology Hospital of Southwest Medical University, Luzhou, China

**Keywords:** pleomorphic adenomas, radiomics, machine learning, capsular, computed tomography

## Abstract

**Objectives:**

Computed tomography (CT) imaging of parotid pleomorphic adenoma (PA) has been widely reported, nonetheless few reports have estimated the capsule characteristics of PA at length. This study aimed to establish and validate CT-based intratumoral and peritumoral radiomics models to clarify the characteristics between parotid PA with and without complete capsule.

**Methods:**

In total, data of 129 patients with PA were randomly assigned to a training and test set at a ratio of 7:3. Quantitative radiomics features of the intratumoral and peritumoral regions of 2 mm and 5 mm on CT images were extracted, and radiomics models of Tumor, External2, External5, Tumor+ External2, and Tumor+External5 were constructed and used to train six different machine learning algorithms. Meanwhile, the prediction performances of different radiomics models (Tumor, External2, External5, Tumor+External2, Tumor+External5) based on single phase (plain, arterial, and venous phase) and multiphase (three-phase combination) were compared. The receiver operating characteristic (ROC) curve analysis and the area under the curve (AUC) were used to evaluate the prediction performance of each model.

**Results:**

Among all the established machine learning prediction radiomics models, the model based on a three-phase combination had better prediction performance, and the model using a combination of intratumoral and peritumoral radiomics features achieved a higher AUC than the model with only intratumoral or peritumoral radiomics features, and the Tumor+External2 model based on LR was the optimal model, the AUC of the test set was 0.817 (95% CI = 0.712, 0.847), and its prediction performance was significantly higher (*p* < 0.05, DeLong’s test) than that with the Tumor model based on LDA (AUC of 0.772), the External2 model based on LR (AUC of 0.751), and the External5 model based on SVM (AUC of 0.667). And the Tumor+External2 model based on LR had a higher AUC than the Tumor+External5 model based on LDA (AUC = 0.817 vs. 0.796), but no statistically significant difference (*P* = 0.667).

**Conclusion:**

The intratumoral and peritumoral radiomics model based on multiphasic CT images could accurately predict capsular characteristics of parotid of PA preoperatively, which may help in making treatment strategies before surgery, as well as avoid intraoperative tumor spillage and residuals.

## 1 Introduction

Pleomorphic adenoma (PA) is the most prevalent benign tumor of the parotid neoplasm, accounting for approximately 66.7% of all parotid tumors (1, 2). Although these slow-growing tumors are often considered low-risk, PAs still have a relatively high risk of malignant transformation and recurrence (1). Previous studies showed that capsular characteristics and surgical approach are the most likely reasons for recurrence (3, 4). The capsule characteristics refer to the appearance of the outer layer or capsule surrounding pleomorphic adenoma of the parotid gland. A well-defined capsule is necessary before surgery as it helps complete tumor removal during surgery, decreasing the rate of recurrence. Thus, accurate preoperative assessment of the capsular characteristics of parotid pleomorphic adenoma is essential for evaluating treatment decisions.

However, there is still no non-invasive, clinically applicable approach for preoperative assessment of capsular characteristics. Fine-needle aspiration cytology (FNAC) can not assess the capsular characteristics. Moreover, preoperative imaging for pleomorphic adenoma of the parotid glands includes ultrasound (US), computed tomography (CT) and magnetic resonance imaging (MRI). Due to US examination being easily affected by the adjacent bone and tumor location, its diagnostic efficacy is limited, so CT and MRI examinations are widely used in clinical practice (5, 6). Previous research has shown computed tomography (CT) imaging features and the histopathology of PAs were poor consistency, even by experienced radiologists to assess the capsular characteristics of PAs (7). And some studies have referred to the capsule of pleomorphic adenomas on MR imaging (8, 9), and indicated that a capsule completely surrounding the tumor has a high positive predictive value for the diagnosis of pleomorphic adenoma (8). However, in cases of pleomorphic adenomas with incomplete capsules, the margin of the lesion is unclear, which may lead to misdiagnosis. Therefore, there is an urgent need to develop more efficient and non-invasive assessments to aid in the preoperative evaluation of the capsular characteristics of parotid PAs.

Radiomics is a relatively new concept that analyzes and extracts quantitative data from medical images, which introduces a new way to mine valuable information contained in the images (10–12). And the feasibility of radiomics as a non-invasive approach has been demonstrated by its wide application in the early differential diagnosis and prognosis evaluation in multiple solid tumors (13, 14). Recently, due to its excellent performance in oncological applications, radiomics has been applied in preoperative identifying different pathological types of parotid tumors. However, those previous radiomics studies mainly focused on the primary tumor area alone (15–18), whereas little was known about the role of peritumoral radiomics features, which were likely to provide valuable but easily overlooked information about parotid tumors.

In this context, we hypothesized that peritumoral radiomics features may offer useful information for the possible infiltration of tumor toward normal tissue, which would be helpful for clinical decision. Thus, we aimed to explore the potential of radiomic features of the intratumoral and peritumoral radiomics features on CT images to preoperative predict the capsular characteristics of the parotid PA.

## 2 Materials and methods

### 2.1 Patients

**FIGURE 1:**
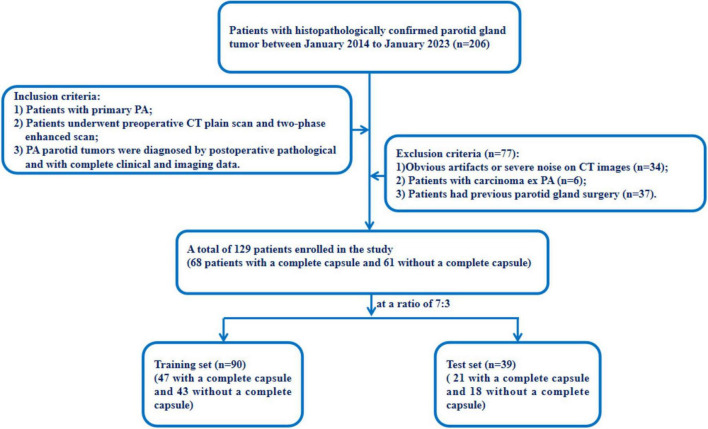
Flowchart for selecting the study population.

This retrospective study was approved by the ethics committees of our hospital (approval number: K2023-414). The requirement for informed consent was waived owing to the retrospective nature of the study. The data of 129 patients with PA who underwent parotid surgery in our hospital from January 2014 to January 2023 were included in the Study. The inclusion criteria were as follows: (1) they were diagnosed with PA through surgical pathology; (2) they underwent plain CT and two-phase enhanced scans before receiving any treatment; (3) patients with primary PA. The exclusion criteria were as follows: (1) they were diagnosed with carcinoma ex PA; (2) images with severe noise or evident artifacts on CT images; (3) patients had previous parotid gland surgery. Figure 1 illustrates the workflow of our study.

### 2.2 The reference standard for capsular characteristics

The study divided patients into two groups: a complete capsule group and a incomplete capsule group. The incomplete capsule group included those patients with PAs who displayed any of the following capsular characteristics (19):

① “incomplete capsule” indicates partial absence of the encapsulation;
② “pseudopodia” indicates tumor nodules are separated by fibrous tissue but remain in contact with the main tumor capsule;
③ “capsule invasion” means that tumor tissue infiltration not separated from the main tumor mass;
④ “satellite nodules” indicates nodules separated from the main tumor by fat tissue or salivary gland, and they located adjacent to the main tumor mass but not connected to it.

All patients in this study underwent complete tumor resection. For the PA patients in the complete capsule group, the diagnosis was based on surgical and pathological reports. They were assigned to this group when the surgeon verified that they had well-defined borders, and the pathologist verified the integrity of the capsule. For patients in the incomplete capsule group, the diagnosis was made based on postoperative pathological results, which included the evaluation of its completeness as well as other capsular characteristics such as satellite nodules, pseudopodia, and capsule invasion.

### 2.3 Image acquisition

Using multi-slice spiral CT equipment, each patient underwent multi-phase scanning, including plain scanning phase, arterial scanning phase, and venous scanning phase. The CT images were stored in the Digital Imaging and Communications in Medicine (DICOM) format. The acquisition parameters of the above different devices are introduced in detail in Supplementary Table 1.

### 2.4 Image segmentation

The CT images of those patients were stored in DICOM format using standard soft tissue settings: window width of 400 HU and window level of 40 HU. Blinded to the histopathological results of the patients, two radiologists (with 3 and 5 years of clinical diagnostic experience) used the ITK-SNAP software (version 3.8.0^1^) to segment the region of interest (ROI) manually. The tumors were delineated along the margins layer-by-layer on axial multi-phase CT images, eliminating the vessels, bone, and normal adjacent tissue. When multiple lesions were found in the parotid gland, the largest lesion with a confirmed pathology was selected for analysis. The intra- and inter-observer reproducibility were evaluated by the intraclass correlation coefficient (ICC). The segmentation was executed independently by radiologist-A and radiologist-B during the same period to evaluate inter-observer agreement of extracted radiomics features. Radiologist-A then repeated the same case procedure 1 month later, and an ICC greater than 0.75 indicated good consistency.

**FIGURE 2:**
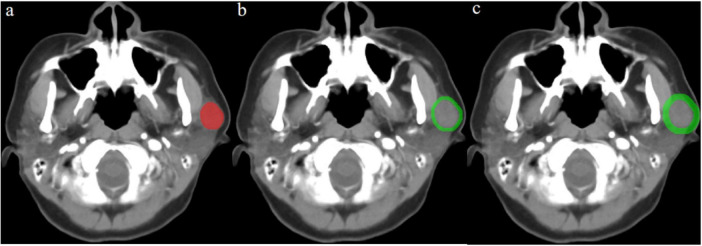
Contrast-enhanced computed tomography (CT) image from a pleomorphic adenoma patient, highlighted regions represent the primary tumor **(a)** and peritumoral region of 2 mm **(b)** and 5 mm **(c)**.

After manual tumor segmentation, 2 mm and 5 mm peritumoral regions were automatically segmented using Python (version 3.7.12^2^) (Figure 2). Next, the bone and air were filtered from the delineation by setting the maximum (400 HU) and minimum (–200 HU) thresholds, and the final ROI border (peritumoral regions) was manually adjusted (20, 21).

### 2.5 Radiomics feature extraction and selection

PyRadiomics in Python was used for feature extraction. In order to reduce the impact of different scanning devices, all CT images were resampled to a voxel spacing of 1 × 1 × 1 mm^3^, and standardization and resampling techniques were applied to preprocess the images and data to ensure the consistency of the CT images between patients. Feature extraction was conducted from five different ROIs—Tumor, External2, External5, Tumor+External2, and Tumor+External5— for each patient using the PyRadiomics Radiomics Feature Extractor toolbox. Each segmented region obtained 1,288 radiomic features, including the first-order (252), gray-level co-occurrence matrix (308), gray-level run-length matrix (224), and gray-level size zone matrix (224), gray-level dependence matrix (196) and neighboring gray-tone difference matrix (70). These algorithms for obtaining radiomics features were referenced from the image biomarker standardization initiative (IBSI) (22).

The radiomics features dimensionality reduction and selection in the training set were as follows: firstly, analysis of variance (ANOVA) was performed on the extracted features to select statistically significant features with ICC scores > 0.9. Secondly, We decreased the dimensionality of the feature space by assessing the similarity of each feature pair and removing one of the features if the Pearson correlation coefficient (PCC) value was higher than 0.95. Ultimately, features with non-zero coefficients were selected using the least absolute shrinkage and selection operator (LASSO) regression model with 10-fold cross-validation, which was described in Supplementary Appendix 1.

### 2.6 Model construction and evaluation

Based on the radiomics features extracted from the plain scan, arterial and venous phase CT images, our study built and validated six machine learning algorithms, including support vector machine (SVM), logistic regression (LR), extreme gradient boosting (XGBoost), linear discriminant analysis (LDA), random forest (RF) and decision tree (DT). For each region (Tumor, External2, External5, Tumor+External2, and Tumor+External5), by combining each machine learning algorithm with 15 different feature sets, 90 models were established. And 10-fold cross-validation on the training set was used to identify the hyperparameters of each model.

We evaluated the diagnostic performance of those models by comparing the area under the curve (AUC) of the receiver operating characteristic curve (ROC), accuracy, positive prediction value (PPV), sensitivity, specificity, and negative prediction value (NPV). Then, the best radiomics model and scanning phase were obtained.

### 2.7 Statistical analysis

All statistical analyses were performed using PyRadiomics in Python (version 3.7.12; see text footnote 2), and SPSS (version 26.0; IBM, Armonk, NY, United States) software. Comparisons between sets were performed using the Student’s *t*-test or the Mann–Whitney U test for continuous variables and the χ^2^ or Fisher exact test for categorical variables. Besides, the “sklearn” packages were used for plotting the curves of the ROC. Two-sided *p* < 0.05 was deemed statistically significant for all statistical tests.

## 3 Results

### 3.1 The population and radiological features of patients

The details of the patient’s clinical and radiological characteristics are shown in Table 1. No significant statistical differences in characteristics were found between the training and test sets in terms of sex, age, smoking, drinking, symptom, shape, margin, density, cystic areas, enhanced uniformity, except for the max diameter, whose *p*-value in the test set were < 0.05. Furthermore, univariate and multivariate logistic regression were used to select the independent clinical predictors of patients, however, we found no significant clinical predictors in this study.

**TABLE 1:** Clinical and computed tomography (CT) morphological characteristics of patients in the training and test sets.

Variables	Training set (*n* = 90)			Test set (*n* = 39)		
	**PA with complete capsule (*n* = 43)**	**PA without complete capsule (*n* = 47)**	***P***	**PA with complete capsule (*n* = 43)**	**PA without complete capsule (*n* = 47)**	***P***
Age^b^ (years)	44.65 ± 16.32	45.96 ± 12.88	0.67	44.27 ± 18.76	48.25 ± 16.57	0.47
Max-diameter^b^ (cm)	2.1 ± 60.68	2.21 ± 0.71	0.71	2.31 ± 0.57	1.67 ± 0.49	< 0.01*
Sex^a^ (F/M)	25/18	30/17	0.58	9/9	7/14	0.29
Smoking^a^ (yes/no)	14/29	9/38	0.15	5/13	8/13	0.50
Drinking^a^ (Yes/No)	7/36	13/43	0.20	4/14	9/12	0.18
Symptoms^a^ (with/without)	5/38	7/40	0.65	1/17	1/20	0.91
Shape^a^ (round/non-round)	9/34	15/32	0.24	3/15	6/15	0.38
Margin^a^ (clear/unclear)	40/3	46/1	0.29	17/1	20/1	0.91
Density^a^ (homogeneous/heterogeneous)	20/23	29/18	0.15	11/7	12/9	0.80
Cystic areas^a^ (with/without)	16/27	19/28	0.75	3/15	8/13	0.15
Enhancement degree^a^ (slight/moderate/obvious)	1/12/30	4/22/21	0.02*	1/10/7	4/10/7	0.37
Enhanced uniformity^a^ (yes/no)	18/25	15/32	0.33	9/9	8/13	0.46

*Represents *p* < 0.05.

^a^Categorical data are presented as numbers (*n*).

^b^Quantitative data are presented as means (standard deviations) or medians (quartiles), *p-*value was calculated using the independent samples *t*-test or Mann–Whitney U test. *p*-value was calculated with the χ^2^ or Fisher’s exact test. PA, pleomorphic adenoma; F, female; M, male.

### 3.2 Radiomic signature models and performances

A total of 1,288 radiomics features were extracted from each region; therefore, 6,440 radiomics features were extracted from images of each scanning phase. Then, five radiomics signatures were established based on Tumor, External2, External5, Tumor+External2, and Tumor+External5. Then, six machine learning methods were used to establish 120 radiomics models in the arterial phase, venous phase, and plain phase, as well as a three-phase combination. The results of different feature screening methods in each phase are shown in Supplementary Tables 2–4.

**FIGURE 3:**
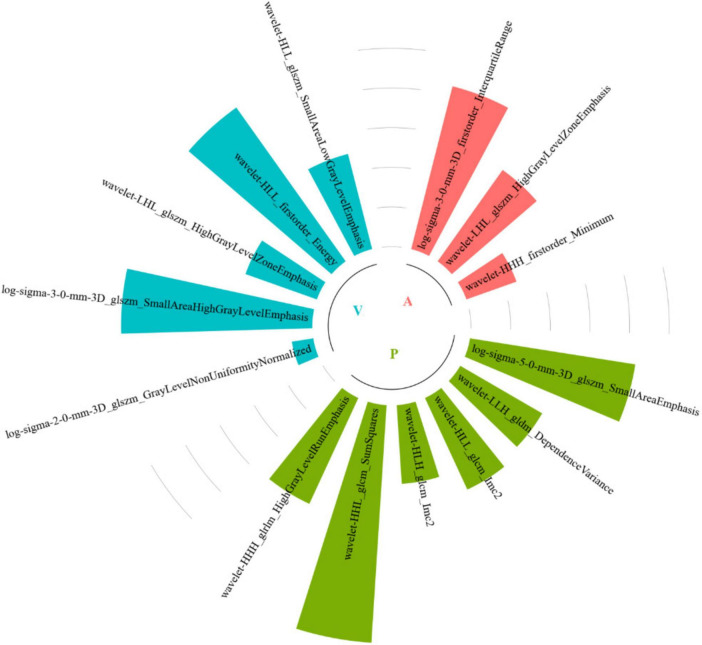
Radiomics feature selection results.

**FIGURE 4:**
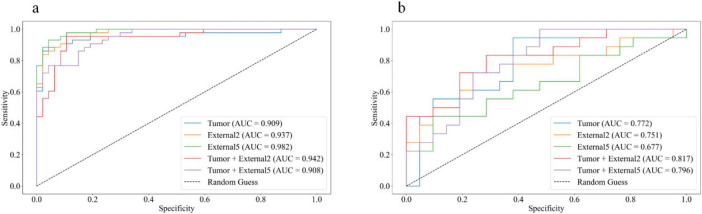
The receiver operating characteristic (ROC) curves of the top performing models based on three-phase combination in the training set **(a)** and test sets **(b)**.

**FIGURE 5:**
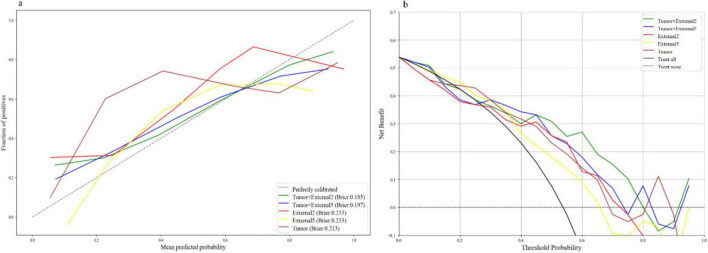
The calibration curves **(a)** and DCA curves **(b)** of the top performing models based on three-phase combination in the test set.

Among all the established machine learning prediction radiomics models, the model based on a three-phase combination had better prediction performance, and the model using a combination of intratumoral and peritumoral radiomics features achieved a higher AUC than the model with only intratumoral or peritumoral radiomic features, which was presented in Table 2. The 14 selected features in the Tumor+External2 model were shown in Figure 3. And the Tumor+External2 model based on LR was the optimal model, the AUC of the test set was 0.817 (95% CI = 0.712, 0.847), and its prediction performance was significantly higher than that with the Tumor model based on LDA (AUC = 0.772, *P* = 0.004), the External2 model based on LR (AUC = 0.751, *P* = 0.032), and the External5 model based on SVM (AUC = 0.667, *P* = 0.018). And the Tumor+External2 model based on LR had a higher AUC than the Tumor+External5 model based on LDA (AUC = 0.817 vs. 0.796), but no statistically significant difference (*P* = 0.667). Figure 4 depicts the ROC curves of the top performing models based on three-phase combination in the training set (a) and test sets (b). The Calibration curves (a) and DCA curves (b) of the top performing models based on three-phase combination in the test set are shown in Figure 5.

**TABLE 2:** Diagnostic performance of different feature screening methods in venous phase.

Model/classifiers	Cohort	AUC (95% CI)	Accuracy	Sensitivity	Specificity	NPV	PPV
**Three-phase combination**							
**SVM**							
Tumor	Training	0.975 (0.950–0.986)	0.944	0.884	1.000	1.000	0.904
	Test	0.720 (0.643–0.779)	0.641	0.611	0.667	0.611	0.667
External2	Training	0.964 (0.949–0.986)	0.967	0.953	0.979	0.976	0.958
	Test	0.614 (0.567–0.688)	0.513	0.500	0.524	0.474	0.550
External5	Training	0.982 (0.928–0.974)	0.911	0.907	0.915	0.907	0.915
	Test	0.677 (0.571–0.700)	0.590	0.556	0.619	0.556	0.619
Tumor+External2	Training	0.940 (0.922–0.969)	0.944	0.977	0.915	0.913	0.977
	Test	0.807 (0.722–0.841)	0.744	0.722	0.762	0.722	0.762
Tumor+External5	Training	0.979 (0.951–0.988)	0.978	0.977	0.979	0.977	0.979
	Test	0.757 (0.662–0.828)	0.564	0.556	0.571	0.526	0.600
**LR**							
Tumor	Training	0.958 (0.890–0.945)	0.900	0.884	0.915	0.905	0.896
	Test	0.749 (0.573–0.793)	0.539	0.444	0.619	0.500	0.565
External2	Training	0.937 (0.916–0.963)	0.900	0.907	0.894	0.886	0.913
	Test	0.751 (0.602–0.780)	0.641	0.667	0.619	0.600	0.684
External5	Training	0.937 (0.867–0.926)	0.867	0.837	0.894	0.878	0.857
	Test	0.606 (0.484–0.656)	0.436	0.556	0.333	0.417	0.467
Tumor+External2	Training	0.942 (0.876–0.935)	0.911	0.930	0.894	0.889	0.933
	Test	0.817 (0.712–0.847)	0.722	0.760	0.762	0.643	0.640
Tumor+External5	Training	0.937 (0.911–0.957)	0.889	0.884	0.894	0.884	0.894
	Test	0.762 (0.672–0.819)	0.667	0.611	0.714	0.647	0.682
**LDA**							
Tumor	Training	0.909 (0.845–0.939)	0.889	0.884	0.894	0.884	0.894
	Test	0.772 (0.567–0.786)	0.692	0.556	0.810	0.714	0.680
External2	Training	0.916 (0.853–0.955)	0.878	0.873	0.915	0.900	0.860
	Test	0.683 (0.534–0.751)	0.615	0.500	0.714	0.600	0.625
External5	Training	0.873 (0.836–0.912)	0.844	0.791	0.894	0.872	0.824
	Test	0.616 (0.472–0.658)	0.513	0.389	0.619	0.467	0.542
Tumor+External2	Training	0.871 (0.858–0.919)	0.844	0.860	0.830	0.822	0.867
	Test	0.815 (0.698–0.857)	0.641	0.756	0.714	0.625	0.652
Tumor+External5	Training	0.908 (0.840–0.930)	0.844	0.884	0.809	0.809	0.884
	Test	0.796 (0.626–0.814)	0.692	0.611	0.762	0.688	0.696
**XGBoost**							
Tumor	Training	0.937 (0.884–0.959)	0.956	0.930	0.979	0.976	0.939
	Test	0.743 (0.468–0.753)	0.590	0.556	0.619	0.556	0.619
External2	Training	0.927 (0.906–0.972)	0.956	0.953	0.957	0.953	0.957
	Test	0.632 (0.505–0.657)	0.538	0.556	0.524	0.500	0.579
External5	Training	0.950 (0.891–0.971)	0.967	0.977	0.957	0.955	0.978
	Test	0.646 (0.424–0.634)	0.487	0.278	0.667	0.417	0.519
Tumor+External2	Training	0.921 (0.873–0.956)	0.933	0.953	0.915	0.911	0.956
	Test	0.712 (0.607–0.757)	0.667	0.611	0.714	0.647	0.682
Tumor+External5	Training	0.945 (0.908–0.982)	1.000	1.000	1.000	1.000	1.000
	Test	0.725 (0.571–0.801)	0.462	0.389	0.524	0.412	0.500
**RF**							
Tumor	Training	0.806 (0.739–0.883)	0.844	0.814	0.872	0.854	0.837
	Test	0.608 (0.436–0.705)	0.615	0.722	0.524	0.565	0.688
External2	Training	0.905 (0.779–0.888)	0.822	0.814	0.830	0.814	0.830
	Test	0.548 (0.416–0.658)	0.436	0.778	0.143	0.438	0.429
External5	Training	0.834 (0.771–0.872)	0.800	0.860	0.745	0.755	0.854
	Test	0.414 (0.320–0.624)	0.487	0.500	0.476	0.450	0.526
Tumor+External2	Training	0.820 (0.754–0.873)	0.822	0.814	0.830	0.814	0.830
	Test	0.716 (0.481–0.756)	0.590	0.611	0.571	0.550	0.632
Tumor+External5	Training	0.925 (0.756–0.894)	0.889	0.884	0.894	0.884	0.894
	Test	0.659 (0.479–0.741)	0.462	0.444	0.476	0.421	0.500
**DT**							
Tumor	Training	0.789 (0.694–0.811)	0.778	0.814	0.745	0.745	0.814
	Test	0.507 (0.371–0.697)	0.539	0.389	0.667	0.500	0.560
External2	Training	0.832 (0.708–0.839)	0.822	0.744	0.894	0.865	0.792
	Test	0.574 (0.372–0.692)	0.513	0.444	0.571	0.471	0.545
External5	Training	0.803 (0.730–0.845)	0.844	0.884	0.809	0.809	0.884
	Test	0.474 (0.346–0.644)	0.462	0.500	0.429	0.429	0.500
Tumor+External2	Training	0.788 (0.697–0.831)	0.800	0.930	0.681	0.727	0.914
	Test	0.509 (0.441–0.714)	0.692	0.722	0.667	0.650	0.737
Tumor+External5	Training	0.853 (0.712–0.849)	0.844	0.930	0.766	0.784	0.923
	Test	0.566 (0.426–0.696)	0.564	0.556	0.571	0.526	0.800

AUC, area under curve; CI, confdence interval; NPV, negative predictive value; PPV, positive predictive value; SVM, support vector machine; LR, logistic regression; LDA, linear discriminant analysis; XGBoost, extreme gradient boosting; RF, random forest; DT, decision tree.

**FIGURE 6:**
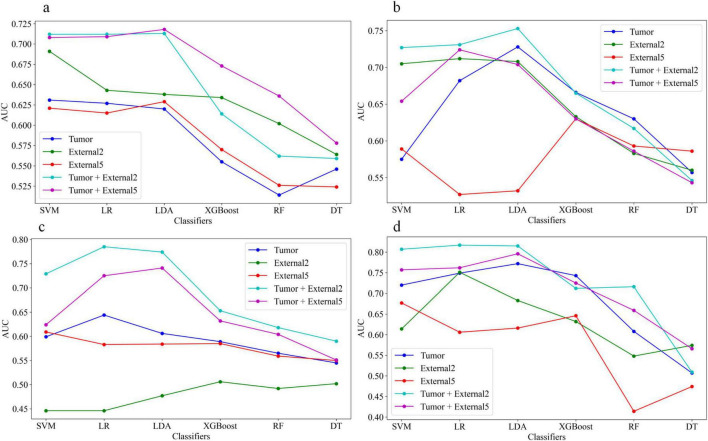
The area under the curve (AUC) values of the five models using six different machine learning algorithm-based models in the test sets, **(a–d)** represent plain phase, arterial phase, venous phase and three-phase combination phase, respectively.

For single phase, the performance of models based on the arterial and venous scan phases were generally better than that in the plain scan phase. Tumor+External2 model based on the venous phase has the highest prediction performance: in the test set (when using the LR classifier), the AUC was 0.785 (95% CI = 0.713, 0.857). The AUC values of the five models using six different machine learning algorithm-based models in the test sets are shown in Figures 6a–d represent plain phase, arterial phase, venous phase and three-phase combination phase, respectively.

## 4 Discussion

In this study, we established and evaluated the ability of multiphasic enhanced CT imaging features from Tumor, External2, External5, Tumor+External2, and Tumor+External5 radiomics features to accurately predict capsular characteristics of PA. We found that regardless of the size of the peritumoral region, radiomics features including the peritumoral signatures were more accurate indicators in the test sets. Specifically, the performance of the Tumor+External2 radiomics model based on the three-phase combination achieved the best performance among all radiomics models in the test sets, with AUC of 0.817 (95% CI = 0.712, 0.847). And the radiomics model based on the LR classifier was superior to the model based on other machine learning algorithms.

Previous radiomics research on PAs has mostly concentrated on the tumor parenchyma, ignoring the tumor-surrounding tissue, while it can reflect the important biological information about the tumor, including its potential for malignant behavior and its interactions with surrounding tissues. Furthermore, the value of tumor-adjacent tissues has been confirmed in recent studies, demonstrating their potential capacity in predicting treatment response, characterizing tumor behavior and evaluating the risk of recurrence (13, 23, 24). However, the application of the peritumoral region in pleomorphic adenoma of the parotid gland has not yet been explored. Besides, the definitions of peritumoral regions have been explored in many previous studies, including distances ranging from 5 mm to 30 mm for lung nodules, 5 mm to 10 mm for breast cancer and 10 mm to 30 mm for malignant brain tumor (13, 25, 26). According to the findings of these studies, the peritumoral area nearest the tumor issue usually provides the highest predictive accuracy.

Unlike previous studies on patients with PAs, which mainly concentrated on intratumoral features and estimated radiomics signatures, we expanded the distance of 2 mm and 5 mm around the tumor and established five radiomic models based on multiphasic enhanced CT imaging to compare their predictive performance. The results indicated that the features extracted from the Tumor+External2 model exhibited the best performance in accurately predicting capsular characteristics of PA, consistent with the results of Li (2). The capsule features appear at the edge of the tumor, and the satellite nodules are more common within a range of 2 mm from the central mass (27), which may contribute to this result. Furthermore, we found that the AUC of the Tumor+External2 model to clarify the capsular characteristics of the parotid PA was higher than that of the Tumor+External5 model. However, there was no statistical significance in this finding. This may suggest that the peritumoral region tissues within 5 mm of the tumor contain valuable information that could identify the encapsulates of PAs.

In addition, our investigation found that the most of the remaining discriminative radiomics features in the Tumor+External2 model were texture features, and this result was consistent with those of previous studies (2, 19), texture features could quantify the inter-voxel relationships in an image and describe microscopic characteristics in CT images of PA. Texture features can reflect the capsular characteristics of the variations in the micro-structures and contain part of pathological characteristics related to the capsule of parotid gland tumor. Furthermore, we applied univariate and multivariate logistic regression to select the independent clinical predictors of patients; however, we found no significant clinical predictors. These results were further evidence that radiomics model was effective and accurate tool for preoperative identification of capsular characteristics of parotid PA.

However, our study faces some limitations. Firstly, the magnetic resonance imaging (MRI) has excellent resolution of the soft tissues, may provide more valuable information compared to CT images. It may be beneficial to explore the potential of MRI-based radiomics features in future studies. Secondly, although the reliability and reproducibility of radiomics feature extraction were satisfactory between the two observers, intratumoral regions were drawn manually to execute image segmentation. Despite using an automatic technique for peritumoral region segmentation, we prefer a fully automatic method that may improve stability and could be applied in future studies. Finally, the sample size in our study was small, and we did not use an independent external validation cohort in this study, thus restricting the generalization ability of our models; thus, larger cohorts, multicentric and external validation are needed for further research and validation.

## 5 Conclusion

In conclusion, CT radiomics features integrating both peritumoral and intratumoral regions could accurately predict capsular morphological characteristics of parotid PA via machine learning models, with obvious advantages compared with conventional image diagnosis, which may provide a valuable tool for preoperative clinical decision-making of patients with parotid PA.

## Data Availability

The raw data supporting the conclusions of this article will be made available by the authors, without undue reservation.

## Footnotes

1
http://www.itksnap.org/

2
http://www.python.org
