# Supplementary material for: The value of multi-phase CT based intratumor and peritumoral radiomics models for evaluating capsular characteristics of parotid pleomorphic adenoma
Source: Front Med (Lausanne). 2025 Apr 22;12:1566555. doi: 10.3389/fmed.2025.1566555 (PMC12054526; doi:10.3389/fmed.2025.1566555)
Supplement: Supplementary file 1 [file Table_1.docx]

**Supplementary Material**

**Supplementary Tables**

Supplementary Table S1. CT Imaging Protocol

Supplementary Table S2. Diagnostic performance of different feature screening methods in arterial phase

Supplementary Table S3. Diagnostic performance of different feature screening methods in plain phase

**Supplementary Appendix**

Supplementary Appendix S1. The least absolute shrinkage and selection operator (LASSO) algorithm

**Supplementary Tables**

**Supplementary Table S1. CT Imaging Protocol**

| CT Imaging Protocol | | | | | | |
| --- | --- | --- | --- | --- | --- | --- |
| CT scanners |  | Discovery  CT750 HD |  | SOMATOM  Definition Flash |  | SOMATOM  Definition Force |
| Tube voltage |  | 100-120 kV |  | 100-120 kV |  | 100 kV |
| Tube current |  | Automatic tube-current |  | Automatic tube-current |  | Automatic tube-current |
| Gantry rotation time |  | 0.6 s |  | 0.5 s |  | 0.28 s |
| Detector collimation |  | 64×0.625mm |  | 128×0.6 mm |  | 128×0.6 mm |
| section thickness |  | 5 mm |  | 5 mm |  | 5 mm |
| section interval |  | 5 mm |  | 5 mm |  | 5 mm |
| Image matrix |  | 512×512 |  | 512×512 |  | 512×512 |
| Contrast agent type |  | Omnipaque |  | Ioversol |  | Ioversol |
| Contrast agent concentration |  | 300mgI/mL |  | 320mgI/mL |  | 320mgI/mL |
| Contrast agent dosage |  | 1.5mL/kg |  | 1.2mL/kg |  | 1.2mL/kg |
| Contrast agent  infused rate |  | 3.0-4.0mL/s |  | 3.0-4.0mL/s |  | 3.0-4.0mL/s |
| Arterial phase scan |  | 30s after the contrast injection |  | 25s after the contrast injection |  | 25s after the contrast injection |
| Venous phase scan |  | 65s after the contrast injection |  | 60s after the contrast injection |  | 60s after the contrast injection |

Note: CT computed tomography.

**Supplementary Table S2.** Diagnostic performance of different feature screening methods in arterial phase

| **Model/Classifiers** | **Cohort** | **AUC [95%CI]** | **Accuracy** | **Sensitivity** | **Specificity** | **NPV** | **PPV** |
| --- | --- | --- | --- | --- | --- | --- | --- |
| **Arterial phase** | | | | | | | |
| SVM | | | | | | | |
| Tumor | Training | 0.745[0.697 -0.793] | 0.689 | 0.651 | 0.723 | 0.683 | 0.694 |
|  | Test | 0.575[0.452 -0.699] | 0.590 | 0.556 | 0.619 | 0.556 | 0.619 |
| External2 | Training | 0.900[0.864 -0.935] | 0.844 | 0.860 | 0.830 | 0.822 | 0.867 |
|  | Test | 0.705[0.601 -0.808] | 0.692 | 0.722 | 0.667 | 0.650 | 0.737 |
| External5 | Training | 0.846[0.794 -0.899] | 0.722 | 0.884 | 0.574 | 0.655 | 0.844 |
|  | Test | 0.589[0.519 -0.659] | 0.590 | 0.444 | 0.714 | 0.571 | 0.600 |
| Tumor + External2 | Training | 0.840[0.794 -0.885] | 0.733 | 0.698 | 0.766 | 0.732 | 0.735 |
|  | Test | 0.727[0.618 -0.836] | 0.641 | 0.667 | 0.619 | 0.600 | 0.684 |
| Tumor + External5 | Training | 0.846[0.800 -0.892] | 0.778 | 0.767 | 0.787 | 0.767 | 0.787 |
|  | Test | 0.654[0.557 -0.751] | 0.718 | 0.722 | 0.714 | 0.684 | 0.750 |
| LR | | | | | | | |
| Tumor | Training | 0.745[0.696 -0.794] | 0.692 | 0.722 | 0.667 | 0.650 | 0.737 |
|  | Test | 0.682[0.652 -0.712] | 0.633 | 0.651 | 0.617 | 0.609 | 0.659 |
| External2 | Training | 0.836[0.800 -0.871] | 0.756 | 0.581 | 0.915 | 0.862 | 0.705 |
|  | Test | 0.712[0.611 -0.812] | 0.590 | 0.611 | 0.571 | 0.550 | 0.632 |
| External5 | Training | 0.759[0.724 -0.793] | 0.689 | 0.651 | 0.723 | 0.683 | 0.694 |
|  | Test | 0.527[0.462 -0.591] | 0.487 | 0.500 | 0.476 | 0.450 | 0.526 |
| Tumor + External2 | Training | 0.758[0.714 -0.802] | 0.700 | 0.791 | 0.617 | 0.654 | 0.763 |
|  | Test | 0.731[0.633 -0.830] | 0.692 | 0.667 | 0.714 | 0.667 | 0.714 |
| Tumor + External5 | Training | 0.755[0.720 -0.791] | 0.622 | 0.860 | 0.404 | 0.569 | 0.760 |
|  | Test | 0.724[0.647 -0.802] | 0.718 | 0.667 | 0.762 | 0.706 | 0.727 |
| LDA | | | | | | | |
| Tumor | Training | 0.688[0.663 -0.713] | 0.644 | 0.698 | 0.596 | 0.612 | 0.683 |
|  | Test | 0.728[0.663 -0.794] | 0.692 | 0.722 | 0.667 | 0.650 | 0.737 |
| External2 | Training | 0.815[0.773 -0.857] | 0.733 | 0.791 | 0.681 | 0.694 | 0.780 |
|  | Test | 0.708[0.607 -0.808] | 0.667 | 0.556 | 0.762 | 0.667 | 0.667 |
| External5 | Training | 0.751[0.709 -0.794] | 0.689 | 0.767 | 0.617 | 0.647 | 0.744 |
|  | Test | 0.532[0.458 -0.606] | 0.513 | 0.500 | 0.524 | 0.474 | 0.550 |
| Tumor + External2 | Training | 0.755[0.717 -0.794] | 0.689 | 0.581 | 0.787 | 0.714 | 0.672 |
|  | Test | 0.753[0.661 -0.845] | 0.667 | 0.833 | 0.524 | 0.600 | 0.786 |
| Tumor + External5 | Training | 0.753[0.719 -0.788] | 0.711 | 0.651 | 0.766 | 0.718 | 0.706 |
|  | Test | 0.704[0.620 -0.788] | 0.692 | 0.722 | 0.667 | 0.650 | 0.737 |
| XGBoost | | | | | | | |
| Tumor | Training | 0.796[0.736 -0.855] | 0.711 | 0.791 | 0.638 | 0.667 | 0.769 |
|  | Test | 0.666[0.545 -0.788] | 0.641 | 0.389 | 0.857 | 0.700 | 0.621 |
| External2 | Training | 0.903[0.856 -0.950] | 0.778 | 0.837 | 0.723 | 0.735 | 0.829 |
|  | Test | 0.633[0.519 -0.748] | 0.564 | 0.444 | 0.667 | 0.533 | 0.583 |
| External5 | Training | 0.889[0.837 -0.940] | 0.878 | 0.860 | 0.894 | 0.881 | 0.875 |
|  | Test | 0.630[0.557 -0.702] | 0.615 | 0.667 | 0.571 | 0.571 | 0.667 |
| Tumor + External2 | Training | 0.871[0.828 -0.914] | 0.756 | 0.535 | 0.957 | 0.920 | 0.692 |
|  | Test | 0.665[0.551 -0.779] | 0.538 | 0.722 | 0.381 | 0.500 | 0.615 |
| Tumor + External5 | Training | 0.869[0.824 -0.913] | 0.733 | 0.535 | 0.915 | 0.852 | 0.683 |
|  | Test | 0.630[0.521 -0.740] | 0.538 | 0.611 | 0.476 | 0.500 | 0.588 |
| RF | | | | | | | |
| Tumor | Training | 0.713[0.643 -0.782] | 0.744 | 0.767 | 0.723 | 0.717 | 0.773 |
|  | Test | 0.630[0.492 -0.768] | 0.718 | 0.667 | 0.762 | 0.706 | 0.727 |
| External2 | Training | 0.795[0.736 -0.853] | 0.678 | 0.465 | 0.872 | 0.769 | 0.641 |
|  | Test | 0.583[0.448 -0.718] | 0.513 | 0.500 | 0.524 | 0.474 | 0.550 |
| External5 | Training | 0.796[0.726 -0.866] | 0.722 | 0.651 | 0.787 | 0.737 | 0.712 |
|  | Test | 0.593[0.470 -0.716] | 0.487 | 0.556 | 0.429 | 0.455 | 0.529 |
| Tumor + External2 | Training | 0.765[0.693 -0.834] | 0.711 | 0.767 | 0.660 | 0.673 | 0.756 |
|  | Test | 0.617[0.493 -0.741] | 0.564 | 0.389 | 0.714 | 0.538 | 0.577 |
| Tumor + External5 | Training | 0.763[0.693 -0.834] | 0.756 | 0.698 | 0.809 | 0.769 | 0.745 |
|  | Test | 0.586[0.469 -0.704] | 0.564 | 0.611 | 0.524 | 0.524 | 0.611 |
| DT | | | | | | | |
| Tumor | Training | 0.693[0.645 -0.741] | 0.656 | 0.512 | 0.787 | 0.688 | 0.638 |
|  | Test | 0.557[0.397 -0.717] | 0.487 | 0.167 | 0.762 | 0.375 | 0.516 |
| External2 | Training | 0.760[0.701 -0.819] | 0.711 | 0.721 | 0.702 | 0.689 | 0.733 |
|  | Test | 0.560[0.395 -0.724] | 0.590 | 0.722 | 0.476 | 0.542 | 0.667 |
| External5 | Training | 0.764[0.690 -0.837] | 0.756 | 0.651 | 0.851 | 0.800 | 0.727 |
|  | Test | 0.586[0.438 -0.734] | 0.615 | 0.333 | 0.857 | 0.667 | 0.600 |
| Tumor + External2 | Training | 0.754[0.668 -0.839] | 0.711 | 0.558 | 0.851 | 0.774 | 0.678 |
|  | Test | 0.546[0.383 -0.709] | 0.744 | 0.667 | 0.810 | 0.750 | 0.739 |
| Tumor + External5 | Training | 0.740[0.681 -0.800] | 0.689 | 0.907 | 0.489 | 0.619 | 0.852 |
|  | Test | 0.543[0.388 -0.698] | 0.513 | 0.500 | 0.524 | 0.474 | 0.550 |

Abbreviations: AUC area under curve, CI confdence interval, NPV negative predictive value, PPV positive predictive value. SVM Support Vector Machine, LR Logistic Regression, LDA linear discriminant analysis, XGBoost extreme gradient boosting , RF random forest, DT decision tree.

**Supplementary Table S3.** Diagnostic performance of different feature screening methods in plain phase

| **Model/Classifiers** | **Cohort** | **AUC [95%CI]** | **Accuracy** | **Sensitivity** | **Specificity** | **NPV** | **PPV** |
| --- | --- | --- | --- | --- | --- | --- | --- |
| **Plain scan** | | | | | | | |
| SVM | | | | | | | |
| Tumor | Training | 0.890[0.850 -0.930] | 0.778 | 0.814 | 0.745 | 0.745 | 0.814 |
|  | Test | 0.631[0.559 -0.702] | 0.564 | 0.500 | 0.619 | 0.529 | 0.591 |
| External2 | Training | 0.877[0.841 -0.914] | 0.833 | 0.884 | 0.787 | 0.792 | 0.881 |
|  | Test | 0.691[0.612 -0.770] | 0.564 | 0.667 | 0.476 | 0.522 | 0.625 |
| External5 | Training | 0.850[0.809 -0.891] | 0.756 | 0.698 | 0.809 | 0.769 | 0.745 |
|  | Test | 0.621[0.513 -0.729] | 0.487 | 0.611 | 0.381 | 0.458 | 0.533 |
| Tumor + External2 | Training | 0.910[0.875 -0.945] | 0.833 | 0.814 | 0.851 | 0.833 | 0.833 |
|  | Test | 0.712[0.664 -0.759] | 0.692 | 0.778 | 0.619 | 0.636 | 0.765 |
| Tumor + External5 | Training | 0.888[0.849 -0.927] | 0.811 | 0.837 | 0.787 | 0.783 | 0.841 |
|  | Test | 0.708[0.645 -0.770] | 0.564 | 0.778 | 0.381 | 0.519 | 0.667 |
| LR | | | | | | | |
| Tumor | Training | 0.831[0.789 -0.874] | 0.678 | 0.698 | 0.660 | 0.652 | 0.705 |
|  | Test | 0.627[0.531 -0.724] | 0.590 | 0.556 | 0.619 | 0.556 | 0.619 |
| External2 | Training | 0.802[0.768 -0.836] | 0.733 | 0.814 | 0.660 | 0.686 | 0.795 |
|  | Test | 0.643[0.566 -0.721] | 0.615 | 0.611 | 0.619 | 0.579 | 0.650 |
| External5 | Training | 0.769[0. 732-0.806] | 0.689 | 0.605 | 0.766 | 0.703 | 0.679 |
|  | Test | 0.615[0.514 -0.716] | 0.538 | 0.556 | 0.524 | 0.500 | 0.579 |
| Tumor + External2 | Training | 0.880[0.836 -0.924] | 0.822 | 0.791 | 0.851 | 0.829 | 0.816 |
|  | Test | 0.712[0.658 -0.766] | 0.615 | 0.611 | 0.619 | 0.579 | 0.650 |
| Tumor + External5 | Training | 0.809[0.779 -0.839] | 0.744 | 0.698 | 0.787 | 0.750 | 0.740 |
|  | Test | 0.709[0.652 -0.766] | 0.667 | 0.667 | 0.667 | 0.632 | 0.700 |
| LDA | | | | | | | |
| Tumor | Training | 0.815[0.763 -0.867] | 0.756 | 0.767 | 0.745 | 0.733 | 0.778 |
|  | Test | 0.620[0.507 -0.734] | 0.590 | 0.333 | 0.810 | 0.600 | 0.586 |
| External2 | Training | 0.780[0.733 -0.826] | 0.756 | 0.791 | 0.723 | 0.723 | 0.791 |
|  | Test | 0.638[0.550 -0.726] | 0.641 | 0.611 | 0.667 | 0.611 | 0.667 |
| External5 | Training | 0.756[0.709 -0.802] | 0.711 | 0.698 | 0.723 | 0.698 | 0.723 |
|  | Test | 0.629[0.530 -0.728] | 0.615 | 0.500 | 0.714 | 0.600 | 0.625 |
| Tumor + External2 | Training | 0.866[0.821 -0.911] | 0.800 | 0.860 | 0.745 | 0.755 | 0.854 |
|  | Test | 0.713[0.637 -0.788] | 0.667 | 0.667 | 0.667 | 0.632 | 0.700 |
| Tumor + External5 | Training | 0.796[0.760 -0.832] | 0.722 | 0.884 | 0.574 | 0.655 | 0.844 |
|  | Test | 0.718[0.632 -0.803] | 0.615 | 0.778 | 0.476 | 0.560 | 0.714 |
| XGBoost | | | | | | | |
| Tumor | Training | 0.905[0.870 -0.939] | 0.800 | 0.953 | 0.660 | 0.719 | 0.939 |
|  | Test | 0.555[0.432 -0.678] | 0.487 | 0.222 | 0.714 | 0.400 | 0.517 |
| External2 | Training | 0.894[0.854 -0.933] | 0.811 | 0.651 | 0.957 | 0.933 | 0.750 |
|  | Test | 0.634[0.569 -0.700] | 0.641 | 0.722 | 0.571 | 0.591 | 0.706 |
| External5 | Training | 0.867[0.829 -0.904] | 0.833 | 0.744 | 0.915 | 0.889 | 0.796 |
|  | Test | 0.570[0.475 -0.666] | 0.538 | 0.611 | 0.476 | 0.500 | 0.588 |
| Tumor + External2 | Training | 0.909[0.866 -0.952] | 0.767 | 0.535 | 0.979 | 0.958 | 0.797 |
|  | Test | 0.614[0.547 -0.681] | 0.564 | 0.667 | 0.476 | 0.522 | 0.625 |
| Tumor + External5 | Training | 0.908[0.866 -0.951] | 0.789 | 0.884 | 0.702 | 0.731 | 0.868 |
|  | Test | 0.673[0.591 -0.755] | 0.487 | 0.444 | 0.524 | 0.444 | 0.524 |
| RF | | | | | | | |
| Tumor | Training | 0.813[0.747 -0.879] | 0.744 | 0.721 | 0.766 | 0.738 | 0.750 |
|  | Test | 0.514[0.368 -0.660] | 0.615 | 0.778 | 0.476 | 0.560 | 0.714 |
| External2 | Training | 0.805[0.748 -0.863] | 0.722 | 0.674 | 0.766 | 0.725 | 0.720 |
|  | Test | 0.602[0.492 -0.712] | 0.564 | 0.444 | 0.667 | 0.533 | 0.583 |
| External5 | Training | 0.773[0.698 -0.848] | 0.722 | 0.744 | 0.702 | 0.696 | 0.750 |
|  | Test | 0.526[0.401 -0.651] | 0.385 | 0.389 | 0.381 | 0.350 | 0.421 |
| Tumor + External2 | Training | 0.816[0.750 -0.883] | 0.767 | 0.767 | 0.766 | 0.750 | 0.783 |
|  | Test | 0.562[0.445 -0.679] | 0.590 | 0.333 | 0.810 | 0.600 | 0.586 |
| Tumor + External5 | Training | 0.817[0.752 -0.882] | 0.744 | 0.791 | 0.702 | 0.708 | 0.786 |
|  | Test | 0.636[0.508 -0.765] | 0.641 | 0.722 | 0.571 | 0.591 | 0.706 |
| DT | | | | | | | |
| Tumor | Training | 0.752[0.696 -0.808] | 0.722 | 0.581 | 0.851 | 0.781 | 0.690 |
|  | Test | 0.546[0.389 -0.702] | 0.385 | 0.278 | 0.476 | 0.313 | 0.435 |
| External2 | Training | 0.766[0.702 -0.829] | 0.700 | 0.767 | 0.638 | 0.660 | 0.750 |
|  | Test | 0.564[0.440 -0.687] | 0.615 | 0.500 | 0.714 | 0.600 | 0.625 |
| External5 | Training | 0.744[0.668 -0.820] | 0.756 | 0.814 | 0.702 | 0.714 | 0.805 |
|  | Test | 0.524 [0.373 -0.674] | 0.487 | 0.389 | 0.571 | 0.438 | 0.522 |
| Tumor + External2 | Training | 0.764[0.697 -0.831] | 0.744 | 0.674 | 0.809 | 0.763 | 0.731 |
|  | Test | 0.559[0.436 -0.682] | 0.462 | 0.444 | 0.476 | 0.421 | 0.500 |
| Tumor + External5 | Training | 0.765[0.704 -0.826] | 0.711 | 0.814 | 0.617 | 0.660 | 0.784 |
|  | Test | 0.578[0.440 -0.717] | 0.615 | 0.500 | 0.714 | 0.600 | 0.625 |

Abbreviations: AUC area under curve, CI confdence interval, NPV negative predictive value, PPV positive predictive value. SVM Support Vector Machine, LR Logistic Regression, LDA linear discriminant analysis, XGBoost extreme gradient boosting , RF random forest, DT decision tree.

**Supplementary Table S4.** Diagnostic performance of different feature screening methods in venous phase

| **Model/Classifiers** | **Cohort** | **AUC [95%CI]** | **Accuracy** | **Sensitivity** | **Specificity** | **NPV** | **PPV** |
| --- | --- | --- | --- | --- | --- | --- | --- |
| **Venous phase** | | | | | | | |
| SVM | | | | | | | |
| Tumor | Training | 0.858[0.810 -0.907] | 0.744 | 0.930 | 0.574 | 0.667 | 0.900 |
|  | Test | 0.599[0.523 -0.675] | 0.538 | 0.500 | 0.571 | 0.500 | 0.571 |
| External2 | Training | 0.868[0.831 -0.904] | 0.767 | 0.791 | 0.745 | 0.739 | 0.795 |
|  | Test | 0.446[0.373 -0.519] | 0.436 | 0.500 | 0.381 | 0.409 | 0.471 |
| External5 | Training | 0.770[0.728 -0.813] | 0.656 | 0.953 | 0.383 | 0.586 | 0.900 |
|  | Test | 0.609[0.549 -0.669] | 0.538 | 0.667 | 0.429 | 0.500 | 0.600 |
| Tumor + External2 | Training | 0.891[0.846-0.937] | 0.789 | 0.651 | 0.915 | 0.875 | 0.741 |
|  | Test | 0.729[0.631-0.827] | 0.692 | 0.556 | 0.810 | 0.714 | 0.680 |
| Tumor + External5 | Training | 0.815[0.756-0.873] | 0.722 | 0.512 | 0.915 | 0.846 | 0.672 |
|  | Test | 0.624[0.517-0.730] | 0.667 | 0.778 | 0.571 | 0.609 | 0.750 |
| LR | | | | | | | |
| Tumor | Training | 0.752[0.674 -0.830] | 0.611 | 0.535 | 0.681 | 0.605 | 0.615 |
|  | Test | 0.644[0.522 -0.766] | 0.436 | 0.611 | 0.286 | 0.423 | 0.462 |
| External2 | Training | 0.790[0.754 -0.825] | 0.733 | 0.674 | 0.787 | 0.744 | 0.725 |
|  | Test | 0.446[0.357 -0.535] | 0.462 | 0.444 | 0.476 | 0.421 | 0.500 |
| External5 | Training | 0.737[0.704 -0.771] | 0.678 | 0.791 | 0.574 | 0.630 | 0.750 |
|  | Test | 0.583[0.524 -0.643] | 0.538 | 0.389 | 0.667 | 0.500 | 0.560 |
| Tumor + External2 | Training | 0.813[0.776-0.849] | 0.811 | 0.791 | 0.830 | 0.810 | 0.813 |
|  | Test | 0.785[0.713-0.857] | 0.744 | 0.722 | 0.810 | 0.750 | 0.739 |
| Tumor + External5 | Training | 0.749[0.690 -0.808] | 0.756 | 0.767 | 0.745 | 0.733 | 0.778 |
|  | Test | 0.725[0.627 -0.824] | 0.615 | 0.667 | 0.524 | 0.565 | 0.688 |
| LDA | | | | | | | |
| Tumor | Training | 0.746[0.677 -0.815] | 0.644 | 0.674 | 0.617 | 0.617 | 0.674 |
|  | Test | 0.606[0.466 -0.745] | 0.564 | 0.611 | 0.524 | 0.524 | 0.611 |
| External2 | Training | 0.776[0.731 -0.821] | 0.711 | 0.581 | 0.830 | 0.758 | 0.684 |
|  | Test | 0.477[0.366 -0.587] | 0.436 | 0.444 | 0.429 | 0.400 | 0.474 |
| External5 | Training | 0.730[0.693 -0.766] | 0.667 | 0.674 | 0.660 | 0.644 | 0.689 |
|  | Test | 0.584[0.499 -0.669] | 0.513 | 0.389 | 0.619 | 0.467 | 0.542 |
| Tumor + External2 | Training | 0.807[0.770 -0.844] | 0.722 | 0.721 | 0.723 | 0.705 | 0.739 |
|  | Test | 0.774[0.682 -0.865] | 0.744 | 0.833 | 0.667 | 0.682 | 0.824 |
| Tumor + External5 | Training | 0.754[0.707 -0.801] | 0.733 | 0.791 | 0.681 | 0.694 | 0.780 |
|  | Test | 0.741[0.651 -0.831] | 0.769 | 0.778 | 0.762 | 0.737 | 0.800 |
| XGBoost | | | | | | | |
| Tumor | Training | 0.852[0.790 -0.914] | 0.700 | 0.651 | 0.745 | 0.700 | 0.700 |
|  | Test | 0.589[0.464 -0.714] | 0.513 | 0.389 | 0.619 | 0.467 | 0.542 |
| External2 | Training | 0.868[0.827-0.910] | 0.800 | 0.791 | 0.809 | 0.791 | 0.809 |
|  | Test | 0.506[0.427 -0.585] | 0.436 | 0.389 | 0.476 | 0.389 | 0.476 |
| External5 | Training | 0.862[0.814 -0.911] | 0.778 | 0.698 | 0.851 | 0.811 | 0.755 |
|  | Test | 0.585[0.488 -0.682] | 0.667 | 0.500 | 0.810 | 0.692 | 0.654 |
| Tumor + External2 | Training | 0.884[0.829 -0.939] | 0.811 | 0.907 | 0.723 | 0.750 | 0.895 |
|  | Test | 0.653[0.539-0.766] | 0.667 | 0.556 | 0.762 | 0.667 | 0.667 |
| Tumor + External5 | Training | 0.871[0.820 -0.922] | 0.778 | 0.628 | 0.915 | 0.871 | 0.729 |
|  | Test | 0.632[0.532 -0.732] | 0.462 | 0.500 | 0.429 | 0.429 | 0.500 |
| RF | | | | | | | |
| Tumor | Training | 0.734[0.665 -0.802] | 0.689 | 0.581 | 0.787 | 0.714 | 0.673 |
|  | Test | 0.565[0.409 -0.721] | 0.641 | 0.778 | 0.524 | 0.583 | 0.733 |
| External2 | Training | 0.783[0.726 -0.840] | 0.756 | 0.698 | 0.809 | 0.769 | 0.745 |
|  | Test | 0.492[0.378 -0.607] | 0.487 | 0.611 | 0.381 | 0.458 | 0.533 |
| External5 | Training | 0.794[0.736 -0.852] | 0.689 | 0.605 | 0.766 | 0.703 | 0.679 |
|  | Test | 0.559[0.432 -0.687] | 0.564 | 0.667 | 0.476 | 0.522 | 0.625 |
| Tumor + External2 | Training | 0.778[0.703 -0.853] | 0.689 | 0.698 | 0.681 | 0.667 | 0.711 |
|  | Test | 0.618[0.480 -0.756] | 0.615 | 0.500 | 0.714 | 0.600 | 0.625 |
| Tumor + External5 | Training | 0.777[0.701 -0.854] | 0.644 | 0.535 | 0.745 | 0.657 | 0.636 |
|  | Test | 0.604[0.461 -0.748] | 0.538 | 0.611 | 0.476 | 0.500 | 0.588 |
| DT | | | | | | | |
| Tumor | Training | 0.714[0.644 -0.785] | 0.678 | 0.581 | 0.766 | 0.694 | 0.667 |
|  | Test | 0.545[0.406 -0.684] | 0.513 | 0.500 | 0.524 | 0.474 | 0.550 |
| External2 | Training | 0.739[0.676-0.802] | 0.767 | 0.767 | 0.766 | 0.750 | 0.783 |
|  | Test | 0.502[0.381 -0.622] | 0.487 | 0.667 | 0.333 | 0.462 | 0.538 |
| External5 | Training | 0.739[0.675 -0.803] | 0.711 | 0.581 | 0.830 | 0.758 | 0.684 |
|  | Test | 0.550[0.408 -0.693] | 0.641 | 0.722 | 0.571 | 0.591 | 0.706 |
| Tumor + External2 | Training | 0.743[0.667 -0.820] | 0.767 | 0.791 | 0.745 | 0.739 | 0.795 |
|  | Test | 0.590[0.454 -0.726] | 0.590 | 0.556 | 0.619 | 0.556 | 0.619 |
| Tumor + External5 | Training | 0.743[0.663 -0.822] | 0.744 | 0.674 | 0.809 | 0.763 | 0.731 |
|  | Test | 0.551[0.424 -0.678] | 0.641 | 0.722 | 0.571 | 0.591 | 0.706 |

**Supplementary Appendix**

**Supplementary Appendix S1.** The least absolute shrinkage and selection operator (LASSO) algorithm

The LASSO algorithm introduced a penalty term (λ) to the loss function during training and parameter solving. By adjusting λ, the mean square error decreased gradually to its lowest point, representing the optimal parameter. Less influential features had their coefficients reduced to 0, retaining only the most important ones. Features with non-zero coefficients at the optimal λ were then ranked based on the absolute value of the coefficient.
